# Multifunctional Injectable Hydrogel Loaded with Cerium-Containing Bioactive Glass Nanoparticles for Diabetic Wound Healing

**DOI:** 10.3390/biom11050702

**Published:** 2021-05-08

**Authors:** Yue-Hua Chen, Zhou-Feng Rao, Yu-Jie Liu, Xiang-Sheng Liu, Yu-Fei Liu, Lan-Ju Xu, Ze-Qi Wang, Jing-Yue Guo, Lin Zhang, Yun-Sheng Dong, Chun-Xiao Qi, Chao Yang, Shu-Fang Wang

**Affiliations:** 1 Key Laboratory of Bioactive Materials, Ministry of Education, College of Life Sciences, Nankai University, Tianjin 300071, China; 2120191061@mail.nankai.edu.cn (Y.-H.C.); 1811414@mail.nankai.edu.cn (Z.-F.R.); lxs_tianjin@163.com (X.-S.L.); 1120200555@mail.nankai.edu.cn (Y.-F.L.); 1810849@mail.nankai.edu.cn (Z.-Q.W.); 2120191063@mail.nankai.edu.cn (J.-Y.G.); linzhang1994@sina.com (L.Z.); yunshengd@163.com (Y.-S.D.); qichunxiaothu@163.com (C.-X.Q.); 2 Key Laboratory of Molecular Microbiology and Technology, Ministry of Education, College of Life Sciences, Nankai University, Tianjin 300071, China; 2120201047@mail.nankai.edu.cn (Y.-J.L.); yangc20119@nankai.edu.cn (C.Y.); 3 Key Laboratory of Bioactive Materials, Ministry of Education, College of Medicine Sciences, Nankai University, Tianjin 300071, China; xulanju@126.com; 4 Hebei Kerui Biological Pharmaceutical Co., Ltd., Shijiazhuang 050000, China

**Keywords:** diabetic foot ulcers, bioactive glass, cerium, hydrogel, angiogenesis

## Abstract

Diabetic foot wound healing is a major clinical problem due to impaired angiogenesis and bacterial infection. Therefore, an effective regenerative dressing is desiderated with the function of promoting revascularization and anti-bacteria. Herein, a multifunctional injectable composite hydrogel was prepared by incorporation of the cerium-containing bioactive glass (Ce-BG) into Gelatin methacryloyl (GelMA) hydrogel. The Ce-BG was synthesized by combining sol-gel method with template method, which maintained spherical shape, chemical structure and phase constitution of bioactive glass (BG). The Ce-BG/GelMA hydrogels had good cytocompatibility, promoted endothelial cells migration and tube formation by releasing Si ion. In vitro antibacterial tests showed that 5 mol % CeO_2_-containing bioactive glass/GelMA (5/G) composite hydrogel exhibited excellent antibacterial properties. In vivo study demonstrated that the 5/G hydrogel could significantly improve wound healing in diabetic rats by accelerating the formation of granulation tissue, collagen deposition and angiogenesis. All in all, these results indicate that the 5/G hydrogel could enhance diabetic wound healing. Therefore, the development of multifunctional materials with antibacterial and angiogenic functions is of great significance to promote the repair of diabetic wound healing.

## 1. Introduction

The International Diabetes Federation estimated that the global prevalence of diabetes would be 693 million in 2045 [1]. In people diagnosed with diabetes, the lifetime risk of foot ulcers is estimated to be 15% to 25% [2]. It has a high rate of disability and death, seriously affects the patients’ life quality, shortens life expectancy and brings heavy social burden. The rate of death risk within 5 years in patients with diabetic foot ulcers (DFUs) is 2.5 times that in patients without DFUs [3]. Angiogenesis plays a vital role in wound healing, because neovascularization is responsible for transporting nutrients and growth factors and removing wastes from new tissue forming sites [4]. Bioactive glass (BG) has been widely used in hard tissue engineering due to its high bioactivity. Recent studies have shown that BG has the ability to promote angiogenesis and repair soft tissue wounds [5]. BG can be basically divided into three groups, depending on the representative network former oxide present in the formulation, i.e., SiO_2_-based (silicate), B_2_O_3_-based (borate) and P_2_O_5_-based (phosphate) systems [6]. SiO_2_-based BG can release Si ions to promote vascular remodeling and wound healing [7,8].

Infection is an extremely important reason of impaired the healing [9]. Cerium (Ce) is a rare earth element, which is widely used in industrial fields such as catalysts, fuel additives and colored components doped in glass, but its potential role in biomedical field are underestimated [10]. Accumulating evidences have indicated that CeO_2_ has antibacterial activity and the Ce^4+^-rich CeO_2_ shows high antimicrobial activity [10,11]. In addition, BG can be doped with a small amount of trace element oxides, such as zinc, copper, zirconium and iron. It has been found that the release of these cations (so-called therapeutic ions) into physiological body fluids can promote some related biological functions, such as osteogenesis, angiogenesis or antibacterial ability [12]. Silver, copper, zinc and gallium have been incorporated into BG to endow it antibacterial ability [6]. Based on the findings, it is interesting to incorporate CeO_2_ into BG for wound healing.

However, direct contact with BG may adhere to the wound bed, causing laceration and other adverse reactions of wound [13]. Hydrogel is a hydrophilic polymer with intrinsic three-dimensional structure [14], which could release of therapeutic molecules as attractive alternatives to many systems approaches [15,16]. Therefore, the hydrogels loaded with BG can avoid adverse reactions caused by BG alone. The hydrogels can be prepared from various types of synthetic biomaterials comprising polyacrylic acid, polyacrylamide and their derivatives and natural biomaterials including alginate, collagen and chitosan. Here, we choose photocrosslinkable and biodegradable Gelatin methacryloyl (GelMA) as the backbone of hydrogel, which is widely used in tissue engineering and regenerative medicine [17,18].

In this study, we fabricated a cerium-containing bioactive glass (Ce-BG)/GelMA composite injectable hydrogel for diabetic skin wound healing. Ce-BG loaded with various content of Ce was synthesized by sol-gel method combined with template method. The effect of the composite hydrogel on cells were explored by cell adhesion, migration and tube formation assays. The agar plate assay was used to assess the antibacterial activity of the composite hydrogels. Finally, we explored the therapeutic efficacy of the composite hydrogels in a diabetic skin defect model. We assume that the composite hydrogel may have multifunctional activity to inhibit the bacterial growth, enhance angiogenic activity and subsequently accelerate wound healing in diabetic skin wound.

## 2. Materials and Methods

### 2.1. Materials

Tetraethyl orthosilicate (TEOS), triethyl phosphate (TEP), calcium nitrate trihydrate (Ca(NO_3_)_2_•3H_2_O), Cerium nitrate hexahydrate (Ce(NO_3_)_3_•6H_2_O), Dodecylamine (CH_3_(CH2)_11_NH_2_) were purchased from Aladdin Industrial Corporation. Gelatin from porcine skin, Methacrylic anhydride (MA) and Lithium phenyl-2, 4, 6-trimethylbenzoylphosphinate (LAP) were purchased by Sigma (Sigma-Aldrich, St. Louis, Missouri, US). Cell counting kit (CCK-8) was purchased from Beyotime Biotechnology Co. Ltd., (Beijing, China). The Live & Dead^TM^ Viability/Cytotoxicity Assay Kit for Animal Cells (Calcein AM, EthD-1) was purchased by US EverBright, (Jiangsu, China). Hematoxylin and eosin (H&E) was purchased by Shandong Sparkjade Biotechnology Co. Ltd., (Shandong, China). Masson’s Trichrome was purchased by Solarbio Science&technology Co. Ltd., (Beijing, China).

### 2.2. Synthesis and Characterization of BG/Ce-BG

Ce-BG nanoparticles with different Ce contents were synthesized by a sol-gel method combined with template method. Ce-BG was comprised of 75SiO_2_-5P_2_O_5_-(20-m)CaO-mCeO_2_ in mol% (m = 0, 2 or 5), named as 0 Ce-BG, 2 Ce-BG and 5 Ce-BG, respectively. Briefly, to 5 Ce-BG, 4 mL of DDA was added into 80 mL of anhydrous alcohol and 20 mL of deionized water at 40 °C for 10 min. Subsequently, 15.9 mL of TEOS and 1.9 mL of TEP were orderly added and stirred for 30 min. 3.58 g of Ca(NO_3_)_2_•4H_2_O and 2.18 g of Ce(NO_3_)_3_•6H_2_O was added into deionized water and stirred until completely dissolved. Then, the mixed solution of Ca(NO_3_)_2_•4H_2_O and Ce(NO_3_)_3_•6H_2_O were added above solution. The mixture was stirred further for 3 h. After the reaction, the product was centrifuged. The precipitate was then washed thrice with water and ethanol successively. Lastly, the precipitate was lyophilized, followed by calcination at 650 °C for 3 h to get the final product.

The morphology and the size distributions of Ce-BG were performed using transmission electron microscope (TEM, HITACHI HT7700 Exalens, Hitachi, Japan) and dynamic light scattering (DLS, ZETAPALS/BI-200SM, BROOKHAVEN, US). The phase compositions and chemical structure of Ce-BG was tested by X-ray diffraction (XRD, Rigaku SmartLab, Japan) and Fourier transform infrared spectroscopy (FT-IR, TENSOR II, Bruker, Germany). The elements in Ce-BG were confirmed by the energy dispersive spectrometer (EDS) system that equipped to scanning electron microscopy (SEM, SU3500, Hitachi, Japan). The valence states of Ce were identified by X-ray photoelectron spectroscopy (XPS, Thermo Scientific ESCALAB 250Xi, Thermo, Waltham， MA, USA).

### 2.3. Synthesis and Characterization of GelMA

GelMA was synthesized as described previously [19]. In brief, type A Gelatin was dissolved at 10% (*w*/*v*) in PBS at 50 °C. 4 mL MA was added at 0.5 mL/min. After 3 h, the solution was diluted with PBS to stop the reaction. The diluted solution was dialyzed against distilled water at 40 °C for 7 days and changed water twice a day. The resulting solution was lyophilized and stored at −20 °C. ^1^H NMR spectra of GelMA and Gelatin were measured on NMR spectrometer (AVANCE III 400 MHz, Bruker, Switzerland).

### 2.4. Preparation and Characterization of Ce-BG/GelMA Composite Hydrogels

For preparation of Ce-BG/GelMA composite hydrogels, the freeze-dried GelMA foams and LAP (photoinitiator) were dissolved at 10% (*w*/*v*) and 0.1% (*w*/*v*) in PBS at 50 °C and stirred until completely dissolved. Then, Ce-BG powders were added at 1% (*w*/*v*) in the above solution. After stirring for 1 h, the solution was crosslinked by 365 nm UV light for 20 s to form hydrogels. The abbreviations of the hydrogels: GelMA (G), 0 Ce-BG/GelMA (0/G), 2 Ce-BG/GelMA (2/G), 5 Ce-BG/GelMA (5/G).

The surface morphology of the composite hydrogels was observed with a SEM after lyophilizing and spraying with gold. The elements in Ce-BG/GelMA hydrogel was confirmed by the EDS system that equipped to SEM. The hydrogels used for mechanical test were cylindrical, with a diameter of 9.5 mm and a height of 5 mm. The compressive modulus of the composite hydrogels was measured with a universal testing machine (M500-25KN, Testometric, UK) at a constant loading rate of 0.5 mm/min (*n* = 6).

In order to investigate the ion release behavior of the composite hydrogels, 1 g hydrogels were added in 10 mL phosphate buffer solution (PBS) at 37 °C. Then, 8 mL release medium was withdrawn and then replaced with 8 mL fresh PBS solution at the defined time intervals. The concentrations of Si in release medium were analyzed by inductively coupled plasma optical emission spectroscopy (ICP-OES, ICAP 7400, Thermo Fisher, Germany) (*n* = 3).

The swelling capacity of the composite hydrogels was tested by gravimetric method [20]. The hydrogels were immersed in PBS at 37 °C. After 24 h, the hydrogels were taken from the water and surface droplets were removed. The swelling ratio of the hydrogels was calculated according to the formula (1) (*n* = 6):

(1) $$\mathrm{Swelling}\;\mathrm{ratio}=\frac{W-W0}{W0}\times 100\%$$

where *W*0 is the weight of the initial hydrogel, W is the weight of the hydrogel after swelling at 24 h.

Rheological properties of hydrogels were determined by rotational rheometer. The rotor diameter is 20 mm. The test temperature is 37 °C. The fixed angular frequency is 1 rad/s. After testing 3 min, the storage modulus (G’) and the loss modulus (G’’) of the hydrogel were obtained.

### 2.5. Cytocompatibility and Cell Adhesion Assay

Mouse fibroblast L929 (L929) cells and human umbilical vein endothelial cells (HUVECs) were used to evaluate the cytocompatibility of the composite hydrogels. The hydrogels were sterilized under UV for 24 h and soaked at 10 mg/mL in 1640 medium and Endothelial Cell Medium at 37 °C for 24 h, the medium was sterilized by 0.22 μm filter membrane to obtain hydrogel extract. L929 cells and HUVECs were plated in 96-well plates at 3000 cells per well and incubated for 24 h in an incubator, the medium was replaced by fresh medium or hydrogel extract. The medium was changed every other day. After incubation for 1, 3 and 5 days, CCK-8 assay was carried out according to the manufacturer’s instructions. The solution absorbance was measured at wavelength of 450 nm with a microplate reader (iMark, Bio-Rad, US) (*n* = 6).

L929 cells were seeded on the composite hydrogels (diameter of 9.5 mm, height of 2 mm) at 4 × 10^4^ cells (50 μL medium) per well. After 1 and 5 days, the hydrogels were washed with PBS and tested according to the manufacturer’s instruction using the Live/Dead staining reagent. The stained samples were washed with PBS and visualized using confocal laser scanning microscope (LSM710, Zeiss, Germany). The living cells were green and dead cells were red.

L929 cells were seeded on the composite hydrogels (diameter of 9.5 mm, height of 2 mm) at 4 × 10^4^ cells (50 μL medium) per well. After being incubated for 24 h, the hydrogels were washed with PBS and fixed with 4% paraformaldehyde for 2 h. Then, the hydrogels were washed with PBS and dehydrated by soaking in ethanol with a concentration gradient (70%, 80%, 90%, 95% for 5 min, dipped in 100% twice). The morphology of attached cells of the hydrogels were observed by SEM after drying.

### 2.6. Cell migration Assay

HUVECs were seeded into the upper chamber at of 2 × 10^4^ cells per well and incubated for 12 h, the medium of upper and lower chamber was replaced by hydrogel Extract. After incubated for 24 h, the upper chamber was fixed with 4% paraformaldehyde for 30 min. Then, the upper chamber was washed with PBS for three times and stained with a 0.5% crystal violet solution for 20 min. The migrated cells were photographed with microscope (Leica, DM3000, Nussloch, Germany).

### 2.7. Tube Formation Assay

To evaluate the angiogenesis activity of the hydrogels in vitro, 150 μL of Matrigel was added into a well of a 48-well plate at cold temperature. After being incubated at 37 °C for 2 h, 300 μL of each group hydrogel extract that has 3 × 10^4^ HUVECs was seeded into the 48-well plate. At 12 h, tube formation was imaged under inverted light microscope.

### 2.8. Antibacterial Activity Evaluation

To evaluate the antibacterial activity of hydrogels, Gram-negative bacteria (*Escherichia coli, E. coli*) and Gram-positive bacteria (*Staphylococcus aureus, S. aureus*) were selected as model bacteria for the experiment. The hydrogels were sterilized by soaking in 75% ethanol for 30 min and washed by PBS for three times. 1 mL of bacterial suspension and hydrogels were added to every well in 24-well plate. After being incubated at 37 °C at 120 rpm for 24 h, the bacterial suspension was diluted with PBS. The 100 μL of diluted bacterial suspension was pour-plated on the typical agar plates. After incubation for 24 h at 37 °C, the number of colony-forming units was photographed and counted by a digital camera (*n* = 3).

### 2.9. In Vivo Wound Healing in a Diabetic Skin Defect Model

Sprague Dawley (SD) rats (8 weeks, 300–320 g) were induced to diabetic rat through intraperitoneal injection of streptozocin (STZ) at 60 mg/kg [21] and the rat with blood glucose levels above 16.7 mM were regarded as diabetic rat. The diabetic rats were anesthetized by isoflurane and the full-thickness round wound (d = 10 mm) was made on the shaved dorsal side and fixed by stitching with a rubber ring to prevent natural skin contraction. The skin of wounds were treated with five groups as the gauze (C, control), GelMA (G), 0 Ce-BG/GelMA (0/G), 5 Ce-BG/GelMA (5/G). For hydrogel groups, the solution was crosslinked by UV to form hydrogels. The wound was shielded with 3M Tegaderm films and fixed by gauze and plaster after the formation of hydrogel. After 7, 14 and 21 days, the wound was photographed by a digital camera and the wound area was measured by Image J software (*n* = 5).

### 2.10. Histology Analysis

The experimental animals were sacrificed and the wound tissues were obtained and fixed in 4% paraformaldehyde overnight. Then, the harvest samples were dehydrated and embedded in paraffin. 6 μm thick paraffin-embedded tissues were performed using paraffin slicing machine (Leica, RM2245, Nussloch, Germany) and stained with hematoxylin and eosin (H&E) and Masson’s trichrome staining according to manufacturer’s instructions for histology analysis.

### 2.11. Immunofluorescence Analysis

For the immunofluorescence staining, the wound tissues were embedded into optimal cutting temperature (OCT) compound and cut into sections with the thickness of 6 μm by freezing microtome. The sections were fixed in ice-cold acetone and washed by PBS, blocked with 5% goat serum with PBS for 45 min. Then, the sections were incubated with mouse-anti CD31 antibody (NB100-64796SS, Novus) or α-smooth muscle actin (α-SMA) antibody (ab5694, Abcam) at 4 °C overnight and washed by PBS, incubated with a secondary antibody for 2 h. Finally, the sections were counter-stained with DAPI-containing mounting solution and imaged with a fluorescence microscope (Axio Imager Z1, ZEISS, Oberkochen, Germany).

### 2.12. Statistical Analysis

The data were expressed as the means ± standard deviation at least in triplicate. Statistical evaluations were performed using GraphPad Prism 8.0. The statistical significance of the difference was carried out using a Student’s t-test and a one-way ANOVA (* *p* < 0.05, ** *p* < 0.01, *** *p* < 0.001, **** *p* < 0.0001).

## 3. Results

### 3.1. Characterization of Ce-BG

**Figure 1:**
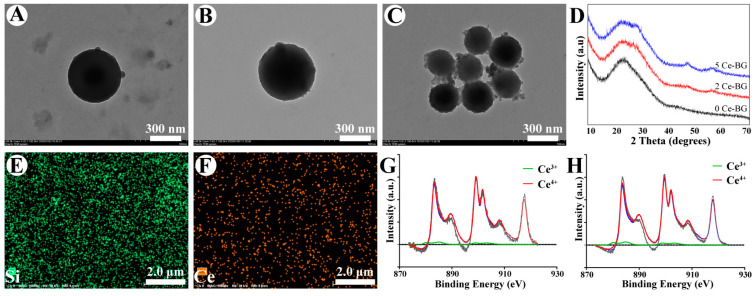
Characterization of Ce-BG. (**A**–**C**) TEM images of 0 Ce-BG, 2 Ce-BG and 5 Ce-BG. (**D**) XRD patterns of 0 Ce-BG, 2 Ce-BG and 5 Ce-BG. (**E**,**F**) EDX mapping images of 5 Ce-BG Si (green), Ce (orange). (**G**,**H**) XPS spectra of Ce in 2 Ce-BG and 5 Ce-BG.

The representative TEM imagines of Ce-BGs (Figure 1A–C) show that Ce-BGs were smooth spherical particles and the 5 Ce-BG had a rough surface. The size distributions of BGs were displayed in Figure S1, the mean particle size of the 0 Ce-BG, 2 Ce-BG and 5 Ce-BG were 362.8 nm, 389.42 and 208.9 nm, respectively. According to the results of TEM imagines and size distributions, we found that 0 Ce-BG and 2 Ce-BG were similar in diameter and the diameter of 5 Ce-BG was smaller than 0 Ce-BG and 2 Ce-BG. XRD patterns of 0 Ce-BG, 2 Ce-BG and 5 Ce-BG (Figure 1D) showed no significant difference and presented strong dispersivity, showing only one peak about at 23° which was characteristic peak of amorphous silicate. Figure S2 showed that the FTIR spectra of 0 Ce-BG, 2 Ce-BG and 5 Ce-BG, the characteristic peaks observed at 1056 cm^−1^, 803 cm^−1^ and 448 cm^−1^ were the symmetric bending of Si-O-Si, symmetric stretching of Si-O and asymmetric stretching of Si-O-Si. The results of XRD and FTIR spectra suggest that the addition of Ce had no influence on the chemical structure and phase constitution of BG. The element distribution in 2 Ce-BG and 5 Ce-BG (Figure 1E,F, Figure S3) analyzed by EDS mapping demonstrated that Si and Ce were uniformly distributed in 2 Ce-BG and 5 Ce-BG, which confirmed the successful preparation of Ce-BG. The valence chemistry of 2 Ce-BG and 5 Ce-BG was measured with XPS as shown in Figure 1G,H, there is mainly Ce^4+^ oxidation state in 2 Ce-BG and 5 Ce-BG, the Ce^4+^ oxidation state laid the foundation for the antibacterial activity [11].

### 3.2. Characterization of Hydrogels

**Figure 2:**
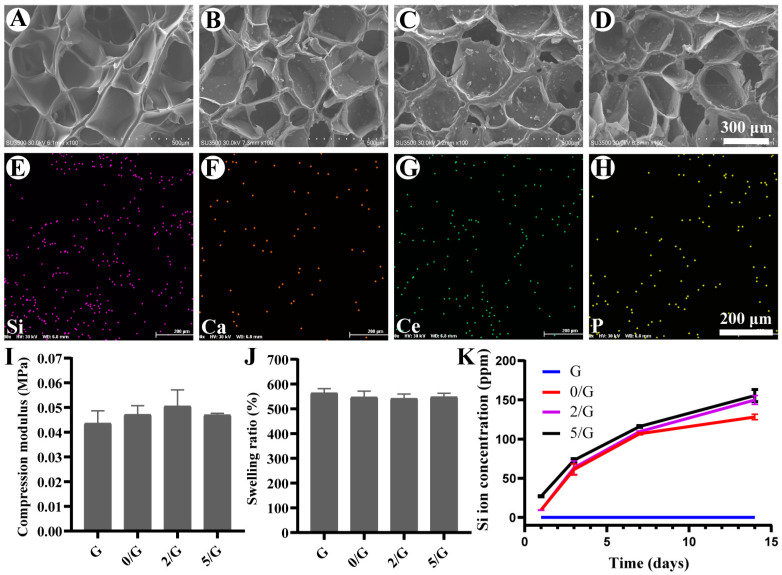
Characterization of hydrogels. (**A**–**D**) SEM images of lyophilized G, 0/G, 2/G and 5/G hydrogels. (**E**–**H**) EDX mapping images of 5/G hydrogel. (**I**) The compression modulus of hydrogels (*n* = 6). (**J**) The swelling ratio of hydrogels (*n* = 6). (**K**) Si ions concentrations released from the hydrogels (*n* = 3).

The ^1^H NMR spectra of Gelatin and Ge1MA are shown in Figure S4. It is found that there are two new peaks at 5.3 ppm and 5.6 ppm in the spectrum of Ge1MA, which belong to the hydrogen peak of C=C in MA, indicating that the methylpropenyl group is successfully grafted onto the molecular chain of Gelatin. As shown in Figure 2A–D, we observed that the lyophilized hydrogels all display porous morphology and the addition of BG did not significantly influence on three-dimensional structure of hydrogel by SEM, but the composite hydrogels had rough surface compared with the G hydrogel. The EDS mapping of 2/G hydrogel and 5/G hydrogel in Figure S5, Figure 2E–H showed the uniform distribution of Si (purple), Ca (orange), Ce (green) and P (yellow) element in the composite hydrogel, indicating that BG was successfully loaded into hydrogels. The compressive modulus of the G, 0/G, 2/G and 5/G hydrogels were 0.043 ± 0.0050, 0.047 ± 0.0035, 0.05 ± 0.0066, 0.047 ± 0.00061 MPa, respectively (Figure 2I). The swelling ratio of the G, 0/G, 2/G and 5/G hydrogels were 563.71 ± 17.78%, 547.11 ± 24.28%, 541.78 ± 17.68%, 548.23 ± 14.48%, respectively (Figure 2J). The addition of BG has negligible influences on hydrogels compressive and swelling properties. The rheological property of hydrogels was shown in Figure S6, when hydrogel precursors were irradiated by UV at 20 s, the G’ instantly increased and formed hydrogel within 20 s. The results of injectable properties presented in Figure S7 show that all groups could be successfully injected. The Si ion release properties of the composite hydrogels in Figure 2K show that they could slowly release Si ion for a long period. The Si ion concentration of 0/G, 2/G and 5/G hydrogels were 9.75 ± 0.10, 9.36 ± 0.37, 27.04 ± 0.73 ppm on day 1 and 128.09 ± 3.54, 149.88 ±5.58, 155.44 ± 7.69 ppm on day 14, the 5/G hydrogel had a higher release rate than other groups could be attributed to the incorporation of other elements into the silica matrix may significantly affect its structural stability [7].

### 3.3. The Cytocompatibility of Hydrogels In Vitro

**Figure 3:**
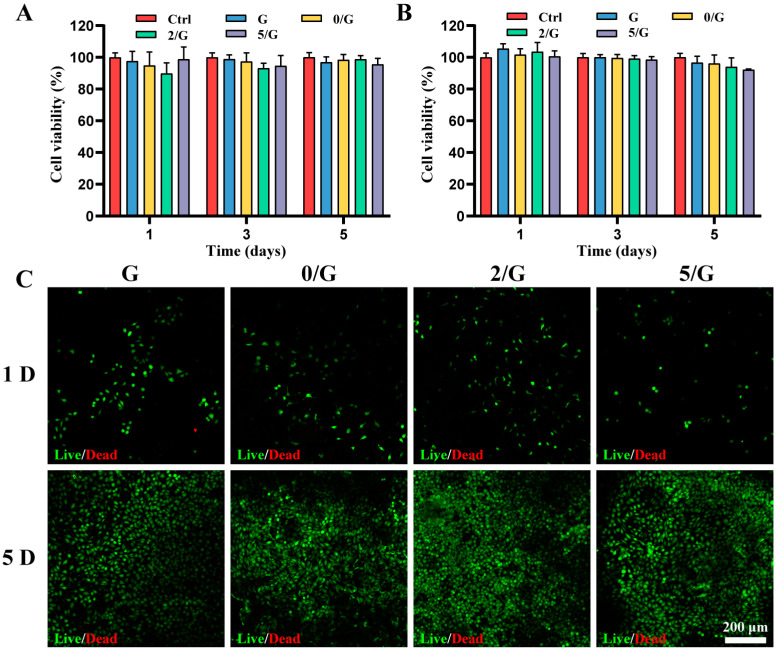
The cytocompatibility of hydrogels. (**A**) Proliferation of HUVECs cultured in different hydrogel extracts (*n* = 6). (**B**) Proliferation of L929 cells cultured in different hydrogel extracts (*n* = 6). (**C**) Live/dead staining images at 1 and 5 days after L929 cells encapsulation in different hydrogels.

Wound dressings should not release any toxic substances through the residual matrix; therefore, proper cytocompatibility is a mandatory feature of ideal dressings. Since the endothelial and fibroblast cells play important roles in repairing tissue defects, HUVECs and L929 cells are chosen to evaluate the cytocompatibility [22]. Figure 3A, B shows the effects of the hydrogels on the proliferation of HUVECs and L929 cells by using CCK-8; there is no statistically significant difference between control and hydrogel groups of cell viability on day 1, 3 and 5. The results of Live/Dead staining are shown in Figure 3C, dead cells were negligible on day 1 and 5, all groups can support both cell growth and proliferation and the number of cells increased with time. The adhesion of cells was observed using SEM (Figure S8), L929 cells could attach and grow well on the hydrogels, which indicated good cell adhesion ability of the hydrogels. Based on the above results, our results indicated that all hydrogels showed good cytocompatibility.

### 3.4. Angiogenic Activity Evaluation

**Figure 4:**
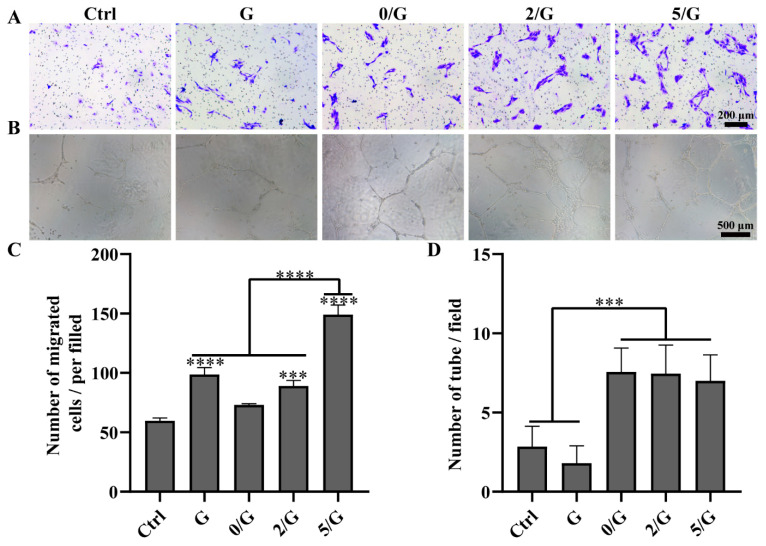
Angiogenic activity evaluation of hydrogels. (**A**) Representative images of HUVECs transwell migration. (**B**) The photographical images of HUVECs tube formation by Matrigel after incubation for 12 h. (**C**) The quantification result of HUVECs migration (*n* = 3). (**D**) The quantification result of HUVECs tube formation (*n* = 3). *** *p* < 0.001, **** *p* < 0.0001.

In the process of wound healing, new capillaries grow into the wound at an amazing speed and produce a large number of new vascular networks, whose density is 2, 3 or even 10 times of that of normal tissue [23]. Endothelial cells are essential cells for blood vessels and play an important role in vascularization [24]. The HUVECs migration and tube formation assays were conducted to assess the angiogenic activity of the hydrogels. The effect of the hydrogels on the migration of HUVECs was shown in Figure 4A,C. It could be easily seen that all the composite hydrogel groups could promote HUVECs migration and the 5/G hydrogel showed the most remarkable effect. A tube-formation assay of the HUVECs were documented and shown in Figure 4B,D. Compared with the control group and G group, all composite hydrogel-treated groups had shown stimulatory effects on HUVECs tube formation.

### 3.5. Antibacterial Activity Evaluation

**Figure 5:**
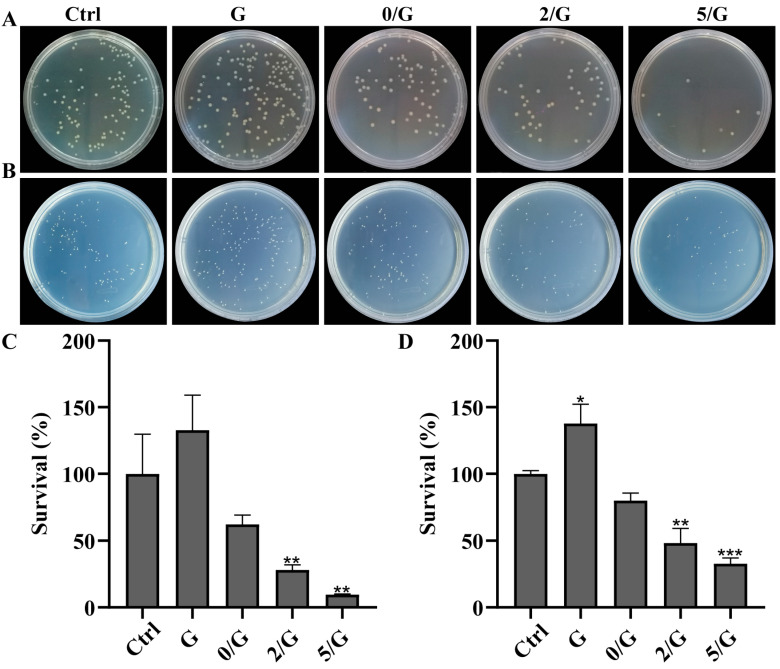
Antibacterial activity evaluation of hydrogels. (**A**,**B**) Photographs of *E. coli* and *S. aureus* grown on agar plates with different treatments, respectively. (**C**) Survival percentage of *E. coli* with different treatments (*n* = 3). (**D**) Survival percentage of *S. aureus* with different treatments (*n* = 3). * *p* < 0.05, ** *p* < 0.01, *** *p* < 0.001.

Figure 5A,B showed the antibacterial activity of the composite hydrogel against *E. coli* and *S. aureus*. The bacterial colony numbers of G hydrogel group was significantly higher than control group. Compared with the control, the composite hydrogels all reduced colony numbers of both types of bacteria, bacterial colony numbers were the least in the agar plate after treatment with 5/G hydrogel. The *E. coli* survival percentage of the G, 0/G, 2/G and 5/G hydrogels were 132.82 ± 26.18%, 62.12 ± 6.94%, 28.08 ± 3.93%, 9.59 ± 0.43%, respectively (Figure 5C). The *S. aureus* survival percentage of the G, 0/G, 2/G and 5/G hydrogels were 137.77 ± 13.45%, 79.81 ± 5.64%, 48.29 ± 10.71%, 32.81 ±4.18%, respectively (Figure 5D). Quantitative results also demonstrated that the 5/G hydrogel group had the lowest survival rate of both types of bacteria.

### 3.6. The Wound Healing Effects on Diabetic Skin Defect Model In Vivo

**Figure 6:**
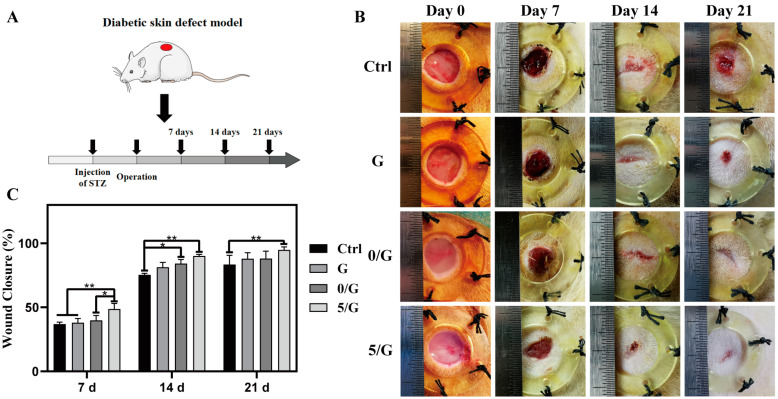
The wound healing after different treatments. (**A**) The flow chart of the whole in vivo experiments. (**B**) Representative images of the skin wound area with different treatments on days 0, 7, 14 and 21. (**C**) Quantification of wound closure rate of different treatment groups (*n* = 5). * *p* < 0.05, ** *p* < 0.01.

Previous studies have shown that the full-thickness skin defect model of diabetic mice can simulate the wound healing process of diabetic patients (Figure 6A) [25]. With the results in vitro, it was demonstrated that the 5/G hydrogel group had better ability to promote cell migration and better antibacterial effect than the 2/G hydrogel group, so we just chose G, 0/G and 5/G hydrogel for the later in vivo experiments. The macroscopic images of diabetic skin wounds with different treatments were shown in Figure 6B. The wound closure time in groups of 0/G and 5/G were faster than the G and control groups, especially in the 5/G group. The quantitative analysis (Figure 6C) confirmed that the wound closure rate of 5/G group was the highest with a wound closure rate of 85.43 ± 6.41% for control group, 88.69 ± 5.13% for G hydrogel group, 88.32 ± 6.65% for 0/G hydrogel group, 94.85 ± 2.33% for 5/G hydrogel group on day 21. Due to the existence of Ce, the systematic biosafety of the hydrogel was extremely concerned. The serum biochemistry tests were used to investigate the influence of the composite hydrogels for kidney and liver functions, it showed that the major indexes of kidney and liver functions of rats stayed in the normal range (Figure S9).

**Figure 7:**
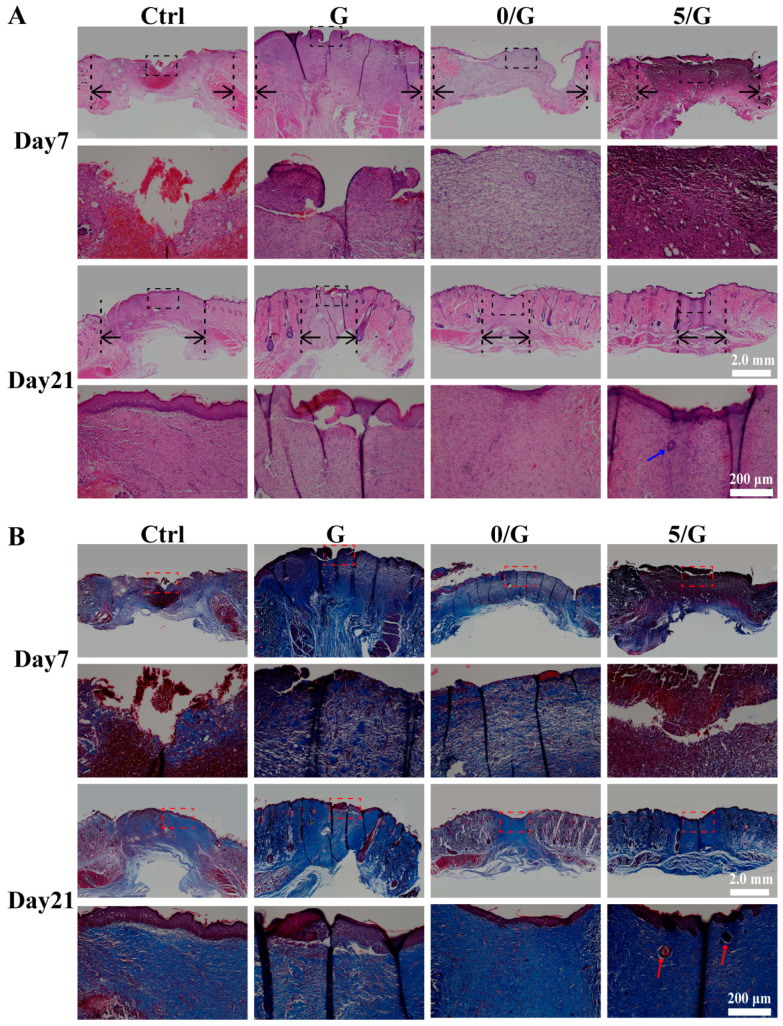
Histology analysis in wounds treated with different hydrogels. (**A**) H&E staining of wound tissue after 7 and 21 days with different treatments. Black arrows indicated microscopic wound edges, blue arrow indicates skin appendages. (**B**) Masson’s trichrome staining of wound tissue after 7 and 21 days with different treatments, red arrow indicates skin appendages.

Normal wound healing is a timely and orderly process of functional recovery and consists of four interconnected and overlapping phases: hemostasis, inflammation, proliferation and tissue remodeling [26,27]. Chronic wounds, such as DFUs, cannot progress continuously in one or more stages of the normal wound healing [28]. To further assess the repair quality of diabetic wound, the regenerated skins were performed by H&E and Masson’s trichrome staining. As shown in Figure 7A, it can be seen that the re-epithelialization rate of 5/G hydrogel group was the highest at day 7 and day 21. Compared to all hydrogel group, a large amount of inflammatory cell infiltration could be seen in the control group on the day 7, this is because hydrogels have the potential to reduce inflammation and potentially regulate local immune responses [17].

In Masson’s trichrome staining (Figure 7B), the blue color was the collagen and the red color was the keratin or muscle fibers [29]. In the 5/G hydrogel group, collagen fibers with higher density were distributed in the wound area at day 7 and day 21, the collagen bundles had smooth surfaces and similar structural orientations to normal skin. The enlarged micrographs further showed the detailed characteristics of the newly regenerated tissue, we could observe the formation of skin appendages of 5/G hydrogel group on the day 21. The results show that 5/G hydrogel group could reconstruct the skin tissue than the other groups.

**Figure 8:**
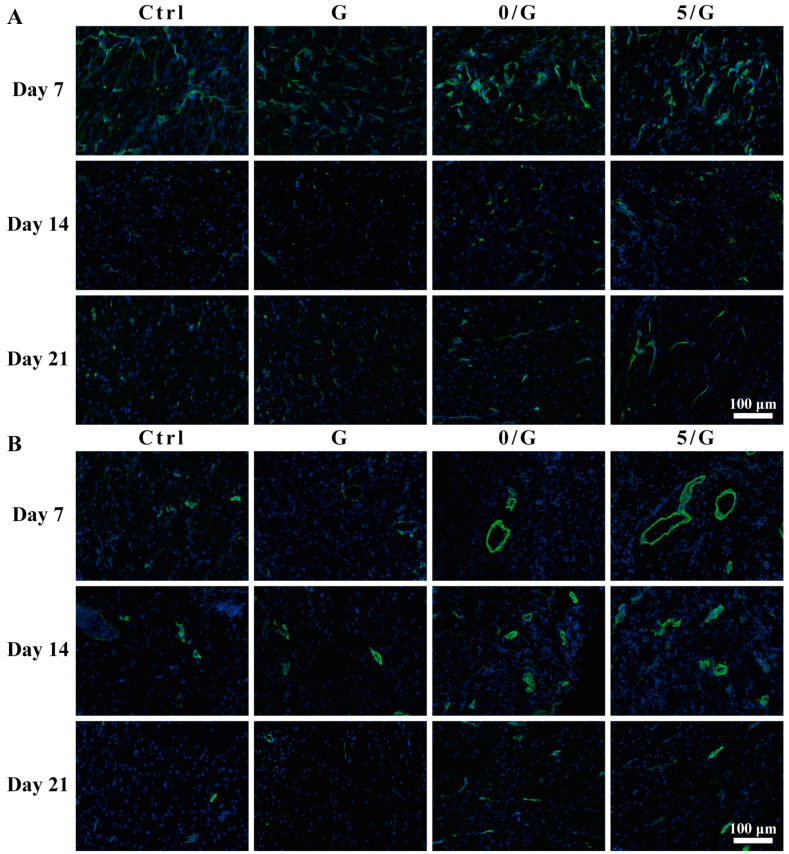
Immunofluorescence staining of wound sections after 7, 14 and 21 days with different treatments. (**A**) Immunofluorescence images of CD31 (green: CD31; blue: nucleus). (**B**) Immunofluorescence images of α-SMA (green: α-SMA; blue: nucleus).

The CD31 and α-SMA immunofluorescence staining were shown in Figure 8A,B. CD31 was highly expressed by endothelial cells and selected to characterize the newly-formed blood vessels, α-SMA was the actin isoform typical of smooth muscle cells and used to characterize the newly formed mature blood vessels [30,31]. The number of CD31 and α-SMA positive cells in the composite hydrogel-treated groups were significantly higher than the control group and GelMA group.

## 4. Discussion

Due to the tough wound healing environment caused by diabetes, the complete and rapid healing of DFUs is challenging [32]. High glucose exposure interferes with the stability of hypoxia-inducible factor-1, leading to the failure of diabetic wound to up-regulate vascular endothelial growth factor (VEGF) in response to soft tissue ischemia, resulting in impaired angiogenesis [24]. Previous studies have shown that BG can release Si ions to promote angiogenesis [13]. In addition, studies have shown that diabetic wound is often the most suitable place for microbial colonization, because of its hyperglycemia, which can easily give rise to wound infection [27]. CeO_2_ can separate the outer membrane from the cytoplasmic membrane to inhibit the growth of bacteria [33]. Zheng et al. have prepared Ag modified mesoporous bioactive glass nanoparticles and proved that it can inhibit bacteria [34]. Li et al. have integrated the monodispersed BGN containing copper into hydrogel, proving that it can enhance the angiogenic ability to diabetic promote wound healing [35]. Therefore, we fabricated the composite hydrogels with the function of promoting revascularization and anti-bacteria for diabetic skin wound healing.

The TEM imaging and the size distribution show that our synthetic Ce-BGs were in the submicron scale (Figure 1A–C, Figure S1). Although the accepted definition of nanomaterials is that materials must have at least one size in the range of 1–100 nm, most reported BGNs show sizes larger than 100 nm and they are actually submicron scale. In addition, single BG nanoparticles (BGNs) smaller than 100 nm tend to agglomerate into larger clusters usually larger than 100 nm [36]. The size ensures that Ce-BGs have special properties of BGNs and without aggregation.

The SEM images show that our hydrogel exhibited a three-dimensional network structure (Figure 2A–D). High porosity in hydrogels is an important physical property, because it has a great influence on cell migration, tissue regeneration, nutrition and oxygen supply and the removal of wastes needed for cell survival [37]. In addition, the porosity of hydrogel has an important effect on the local angiogenesis and the mechanical properties of hydrogels [38]. The swelling ratio and compressive modulus (Figure 2I,J) of the different hydrogels group did not exhibit remarkable changes, which could be due to the low concentrations of the BG. Excellent swelling ability is one of the important conditions to recover scar and promote skin wound healing by providing moist environment [39]. It is also proved that the high swelling ability enables hydrogels to enrich coagulation factors and increase bleeding rate after bleeding [40].

The excellent biocompatibility is mainly attributed to the natural polymer-based hydrogel, which have gained more and more attention owing to their biocompatibility, bioactivity and similarity to native extracellular matrix [41]. Our results (Figure S8) also show that all GelMA based hydrogels have excellent cell adhesion properties. Due to the bioactive motifs of Gelatin, arginine-glycine-aspartic acid (RGD), which can promote the adhesion and growth of different types of cells. In particular, RGD motifs do not contain functional groups that can react with MA; thus, GelMA materials maintained the cell adhesion properties [14,37].

Microbiological studies have shown that bacterial infection is the main reason for delaying the healing of chronic wounds. Many chronic ulcers with bacterial infection take a long time to heal and some even take several years to heal [42]. Therefore, antibacterial activity is a prerequisite for clinical application of hydrogel as wound dressing. In our study, we found that the incorporation of BGs into hydrogel matrix can reduced colony numbers of bacteria, but the effect is not obvious (Figure 5). A relative high pH value is caused by the direct use of BG powders at the wound has antimicrobial effect. We observed that the hydrogel loaded with Ce-BG had antibacterial activity against *E. coli* and *S. aureus* and increased with the increase of Ce content (Figure 5). It has been reported that CeO_2_ can attack bacterial outer membrane proteins with the thiol groups (-SH) reaction through bacterial electron flow and respiration [43]. CeO_2_ also shows the mimic activity of deoxyribonuclease (DNase), which may play a role of cleavage ability by eDNA and dissipate biofilms [44]. The wound dressings with antibacterial activity could prevent the contamination of bacteria to promote wound healing [45].

As shown in Figure 4, the composite hydrogels could promote HUVECs tube formation and migration, which was a challenge for diabetic wounds exposed to hyperglycemia, hypoxia and high oxidative stress [46]. Our further data showed that CD31 and α-SMA immunofluorescence staining in the composite hydrogels group were significantly enhanced compared with G hydrogel and control groups, which confirmed the effect of BGs on angiogenesis in vivo (Figure 8). Previous studies have shown that Si ion in BGs can up-regulate the expression of VEGF and promote vascular remodeling [7,8]. Therefore, in combination with in vivo and in vitro results, our research has successfully demonstrated that the composite hydrogels can indeed promote angiogenesis during wound healing. In the process of neovascularization, oxygen, nutrients, cells and growth factors required for wound healing could infiltrate into the wound site [47].

## 5. Conclusions

In summary, a multifunctional hydrogel has been developed as a wound dressing for diabetic wound. The multifunctional hydrogel was fabricated by GelMA hydrogel containing with Ce-BG. The Ce-BG was synthesized by a sol-gel method combined with template method and have the ability to release Si ion. The three-dimensional structure, swelling ratio and compressive properties of the hydrogels can provide a benefit environment for wound healing. The CCK-8 and Live/Dead staining tests proved that the hydrogels have excellent cell compatibility and the hydrogels could support cell adhesion. The incorporation of Ce-BG into the GelMA hydrogel endowed the composite hydrogel with bioactivity of enhance angiogenic by promoted HUVECs migration, tube formation. The agar plate assay shows the 5/G hydrogel have antibacterial activity against *E. coli* and *S. aureus*. In addition, the 5/G hydrogel could enhance wound healing speed and reconstruct the skin tissue. Overall, our study suggests that the 5/G hydrogel is a promising wound dressing for DFUs.

## Data Availability

All data generated or analyzed during the current study are included in this published article.

## Supplementary Materials

The following are available online at https://www.mdpi.com/2218-273X/11/5/702/s1, Figure S1: Diameter distribution of 0 Ce-BG, 2 Ce-BG and 5 Ce-BG, Figure S2: The FTIR spectra of 0 Ce-BG, 2 Ce-BG and 5 Ce-BG, Figure S3: EDX mapping images of 2 Ce-BG, Figure S4: The ^1^H NMR spectra of GelMA and Gelatin, Figure S5: EDX mapping images of 2/G hydrogel, Figure S6. The rheological property of hydrogels. Figure S7. The injectable properties of hydrogel. Figure S8: L929 cells adhesion on hydrogels, Figure S9: Evaluation of liver and kidney function of rats 21 days after the treatment of the hydrogels.

## References

[1] Cho N.H., Shaw J.E., Karuranga S., Huang Y., da Rocha Fernandes J.D., Ohlrogge A.W., Malanda B. (2018). IDF diabetes atlas: Global estimates of diabetes prevalence for 2017 and projections for 2045. Diabetes Res. Clin. Pract..

[2] Singh N., Armstrong D.G., Lipsky B.A. (2005). Preventing foot ulcers in patients with diabetes. JAMA.

[3] Armstrong D.G., Boulton A.J.M., Bus S.A. (2017). Diabetic foot ulcers and their recurrence. N. Engl. J. Med..

[4] Wu H., Li F., Shao W., Gao J., Ling D. (2019). Promoting angiogenesis in oxidative diabetic wound microenvironment using a nanozyme-reinforced self-protecting hydrogel. ACS Cent. Sci..

[5] Li J., Zhai D., Lv F., Yu Q., Ma H., Yin J., Yi Z., Liu M., Chang J., Wu C. (2016). Preparation of copper-containing bioactive glass/eggshell membrane nanocomposites for improving angiogenesis, antibacterial activity and wound healing. Acta Biomater..

[6] Kaya S., Cresswell M., Boccaccini A.R. (2018). Mesoporous silica-based bioactive glasses for antibiotic-free antibacterial applications. Mater. Sci. Eng. C Mater. Biol. Appl..

[7] Zhang Y., Chang M., Bao F., Xing M., Wang E., Xu Q., Huan Z., Guo F., Chang J. (2019). Multifunctional Zn doped hollow mesoporous silica/polycaprolactone electrospun membranes with enhanced hair follicle regeneration and antibacterial activity for wound healing. Nanoscale.

[8] Kong L., Wu Z., Zhao H., Cui H., Shen J., Chang J., Li H., He Y. (2018). Bioactive injectable hydrogels containing desferrioxamine and bioglass for diabetic wound healing. ACS Appl. Mater. Interfaces.

[9] Falanga V. (2005). Wound healing and its impairment in the diabetic foot. Lancet.

[10] Goh Y.-F., Alshemary A.Z., Akram M., Kadir M.R.A., Huaaain R. (2014). In-vitro characterization of antibacterial bioactive glass containing ceria. Ceram. Int..

[11] Matter M.T., Furer L.A., Starsich F.H.L., Fortunato G., Pratsins S.E., Herrmann I.K. (2019). Engineering the bioactivity of flame-made ceria and ceria/bioglass hybrid nanoparticles. ACS Appl. Mater. Interfaces.

[12] Wang X., Cheng F., Liu J., Smatt J.H., Gepperth D., Lastusaari M., Xu C., Hupa L. (2016). Biocomposites of copper-containing mesoporous bioactive glass and nanofibrillated cellulose: Biocompatibility and angiogenic promotion in chronic wound healing application. Acta Biomater..

[13] Bao F., Pei G., Wu Z., Zhuang H., Zhang Z., Huan Z., Wu C., Chang J. (2020). Bioactive self-pumping composite wound dressings with micropore array modified janus membrane for enhanced diabetic wound healing. Adv. Funct. Mater..

[14] Xiao S., Zhao T., Wang J., Wang C., Du J., Ying L., Lin J., Zhang C., Hu W., Wang L. (2019). Gelatin methacrylate (GelMA)-based hydrogels for cell transplantation: An effective strategy for tissue engineering. Stem. Cell Rev. Rep..

[15] Dimatteo R., Darling N.J., Segura T. (2018). In situ forming injectable hydrogels for drug delivery and wound repair. Adv. Drug Deliv. Rev..

[16] Xue M., Zhao R., Lin H., Jackson C. (2018). Delivery systems of current biologicals for the treatment of chronic cutaneous wounds and severe burns. Adv. Drug Deliv. Rev..

[17] Cheng H., Shi Z., Yue K., Huang X., Xu Y., Gao C., Yao Z., Zhang Y., Wang J. (2021). Sprayable hydrogel dressing accelerates wound healing with combined reactive oxygen species-scavenging and antibacterial abilities. Acta Biomater..

[18] Wang Z., Kumar H., Tian Z., Jin X., Holzman J.F., Menard F., Kim K. (2018). Visible light photoinitiation of cell-adhesive gelatin methacryloyl hydrogels for stereolithography 3D bioprinting. ACS Appl. Mater. Interfaces.

[19] Hong Y., Zhou F., Hua Y., Zhang X., Ni C., Pan D., Zhang Y., Jiang D., Yang L., Lin Q. (2019). A strongly adhesive hemostatic hydrogel for the repair of arterial and heart bleeds. Nat. Commun..

[20] Liang Y., Li Z., Huang Y., Yu R., Guo B. (2021). Dual-dynamic-bond cross-linked antibacterial adhesive hydrogel sealants with on-demand removability for post-wound-closure and infected wound healing. ACS Nano.

[21] Goyal S.N., Reddy N.M., Patil K.R., Nakhate K.T., Ojha S., Pantil C.R., Agrawal Y.O. (2016). Challenges and issues with streptozotocin-induced diabetes—A clinically relevant animal model to understand the diabetes pathogenesis and evaluate therapeutics. Chem. Biol. Interact..

[22] Zhou Q., Kang H., Bielec M., Wu X., Cheng Q., Wei W., Dai H. (2018). Influence of different divalent ions cross-linking sodium alginate-polyacrylamide hydrogels on antibacterial properties and wound healing. Carbohydr. Polym..

[23] DiPietro L.A. (2016). Angiogenesis and wound repair: When enough is enough. J. Leukoc. Biol..

[24] Chen H., Cheng Y., Tian J., Yang P., Zhang X., Chen Y., Hu Y., Wu J. (2020). Dissolved oxygen from microalgae-gel patch promotes chronic wound healing in diabetes. Sci. Adv..

[25] Yu B., He C., Wang W., Ren Y., Yang J., Guo S., Zheng Y., Shi X. (2020). Asymmetric wettable composite wound dressing prepared by electrospinning with bioinspired micropatterning enhances diabetic wound healing. ACS Appl. Biol. Mater..

[26] Malone-Povolny M.J., Maloney S.E., Schoenfisch M.H. (2019). Nitric oxide therapy for diabetic wound healing. Adv. Healthc. Mater..

[27] Xu Z., Han S., Gu Z., Wu J. (2020). Advances and impact of antioxidant hydrogel in chronic wound healing. Adv. Healthc. Mater..

[28] Saleh B., Dhaliwal H.K., Portillo-Lara R., Sani E.S., Abdi R., Amiji M.M., Annabi N. (2019). Local immunomodulation using an adhesive hydrogel loaded with miRNA-laden nanoparticles promotes wound healing. Small.

[29] Guo Z., He J.-X., Mahadevegowda S.H., Kho S.H., Chan-Park M.B., Liu X.W. (2020). Multifunctional glyco-nanosheets to eradicate drug-resistant bacteria on wounds. Adv. Healthc. Mater..

[30] Ouyang J., Ji X., Zhang X., Feng C., Tang Z., Kong N., Xie A., Wang J., Sui X., Deng L. (2020). In situ sprayed NIR-responsive, analgesic black phosphorus-based gel for diabetic ulcer treatment. Proc. Natl. Acad. Sci. USA.

[31] Wei S., Xu P., Yao Z., Cui X., Lei X., Li L., Dong Y., Zhu W., Guo R., Cheng B. (2021). A composite hydrogel with co-delivery of antimicrobial peptides and platelet-rich plasma to enhance healing of infected wounds in diabetes. Acta Biomater..

[32] Lee M., Han S.H., Choi W.J., Chung K.H., Lee J.W. (2016). Hyaluronic acid dressing (healoderm) in the treatment of diabetic foot ulcer: A prospective, randomized, placebo-controlled, single-center study. Wound. Repair. Regen..

[33] Łapa A., Cresswell M., Campbell I., Jackson P., Goldmann W.H., Detsch R., Parsons A., Ahmed I., Boccaccini A.R. (2019). Ga and Ce ion-doped phosphate glass fibres with antibacterial properties and their composite for wound healing applications. J. Mat. Chem. B.

[34] Zheng K., Balasubramanian P., Paterson T.E., Stein R., MacNeil S., Fiorilli S., Vitale-Brovarone C., Shepherd J., Boccaccini A.R. (2019). Ag modified mesoporous bioactive glass nanoparticles for enhanced antibacterial activity in 3D infected skin model. Mater. Sci. Eng. C Mater. Biol. Appl..

[35] Li Y., Xu T., Tu Z., Dai W., Xue Y., Tang C., Gao W., Mao C., Lei B., Lin C. (2020). Bioactive antibacterial silica-based nanocomposites hydrogel scaffolds with high angiogenesis for promoting diabetic wound healing and skin repair. Theranostics.

[36] Zheng K., Boccaccini A.R. (2017). Sol-gel processing of bioactive glass nanoparticles: A review. Adv. Colloid Interface Sci..

[37] Ahmadian Z., Correia A., Hasany M., Figueiredo P., Dobakhti F., Eskandari M.R., Hosseini S.H., Abiri R., Khorshid S., Hirvonen J. (2021). A hydrogen-bonded extracellular matrix-mimicking bactericidal hydrogel with radical scavenging and hemostatic function for pH-responsive wound healing acceleration. Adv. Healthc. Mater..

[38] Annabi N., Nichol J.W., Zhong X., Ji C., Koshy S., Khademhosseini A., Dehghani F. (2010). Controlling the porosity and microarchitecture of hydrogels for tissue engineering. Tissue Eng. Part B Rev..

[39] Amirian J., Zeng Y., Shekh M.I., Sharma G., Stadler F.J., Song J., Du B., Zhu Y. (2021). In-situ crosslinked hydrogel based on amidated pectin/oxidized chitosan as potential wound dressing for skin repairing. Carbohydr. Polym..

[40] Leonhardt E.E., Kang N., Hamad M.A., Wooley K.L., Elsabahy M. (2019). Absorbable hemostatic hydrogels comprising composites of sacrificial templates and honeycomb-like nanofibrous mats of chitosan. Nat. Commun..

[41] Yang R., Liu X., Ren Y., Xue W., Liu S., Wang P., Zhao M., Xu H., Chi B. (2021). Injectable adaptive self-healing hyaluronic acid/poly (γ-glutamic acid) hydrogel for cutaneous wound healing. Acta Biomater..

[42] Elsner J.J., Berdicevsky I., Zilberman M. (2011). In vitro microbial inhibition and cellular response to novel biodegradable composite wound dressings with controlled release of antibiotics. Acta Biomater..

[43] Li X., Qi M., Sun X., Weir M.D., Tay F.R., Oates T.W., Dong B., Zhou Y., Wang L., Xu H.H.K. (2019). Surface treatments on titanium implants via nanostructured ceria for antibacterial and anti-inflammatory capabilities. Acta Biomater..

[44] Liu Z., Wang F., Ren J., Qu X. (2019). A series of MOF/Ce-based nanozymes with dual enzyme-like activity disrupting biofilms and hindering recolonization of bacteria. Biomaterials.

[45] Huang W.C., Ying R., Wang W., Guo Y., He Y., Mo X., Xue C., Mao X. (2020). A macroporous hydrogel dressing with enhanced antibacterial and anti-inflammatory capabilities for accelerated wound healing. Adv. Funct. Mater..

[46] Shiekh P.A., Singh A., Kumar A. (2020). Exosome laden oxygen releasing antioxidant and antibacterial cryogel wound dressing OxOband alleviate diabetic and infectious wound healing. Biomaterials.

[47] Yoon D.S., Lee Y., Ryu H.A., Jang Y., Lee K., Choi Y., Choi W., Lee M., Park K.D., Lee J. (2016). Cell recruiting chemokine-loaded sprayable Gelatin hydrogel dressings for diabetic wound healing. Acta Biomater..

