# Correction: Chen et al. Multifunctional Injectable Hydrogel Loaded with Cerium-Containing Bioactive Glass Nanoparticles for Diabetic Wound Healing. *Biomolecules* 2021, *11*, 702

**DOI:** 10.3390/biom14030283

**Published:** 2024-02-27

**Authors:** Yue-Hua Chen, Zhou-Feng Rao, Yu-Jie Liu, Xiang-Sheng Liu, Yu-Fei Liu, Lan-Ju Xu, Ze-Qi Wang, Jing-Yue Guo, Lin Zhang, Yun-Sheng Dong, Chun-Xiao Qi, Chao Yang, Shu-Fang Wang

**Affiliations:** 1 Key Laboratory of Bioactive Materials, Ministry of Education, College of Life Sciences, Nankai University, Tianjin 300071, China; 2120191061@mail.nankai.edu.cn (Y.-H.C.); 1811414@mail.nankai.edu.cn (Z.-F.R.); lxs_tianjin@163.com (X.-S.L.); 1120200555@mail.nankai.edu.cn (Y.-F.L.); 1810849@mail.nankai.edu.cn (Z.-Q.W.); 2120191063@mail.nankai.edu.cn (J.-Y.G.); linzhang1994@sina.com (L.Z.); yunshengd@163.com (Y.-S.D.); qichunxiaothu@163.com (C.-X.Q.); 2 Key Laboratory of Molecular Microbiology and Technology, Ministry of Education, College of Life Sciences, Nankai University, Tianjin 300071, China; 2120201047@mail.nankai.edu.cn (Y.-J.L.); yangc20119@nankai.edu.cn (C.Y.); 3 Key Laboratory of Bioactive Materials, Ministry of Education, College of Medicine Sciences, Nankai University, Tianjin 300071, China; xulanju@126.com; 4 Hebei Kerui Biological Pharmaceutical Co., Ltd., Shijiazhuang 050000, China

The authors would like to replace Figure 4A of the following published paper [1]. The new Figure 4A is attached below.

In the original publication [1], there was a mistake in Figure 4A. We inadvertently used the same image for the Ctrl group and the G group.

There was an error in the “2.2. Synthesis and Characterization of BG/Ce-BG” section, where the sentence now reading “Ce-BG was comprised of 75SiO_2_-5P_2_O_5_-(20-m)CaO-mCeO_2_ in mol% (*m* = 0, 2 or 5), named as 0 Ce-BG, 2 Ce-BG and 5 Ce-BG, respectively.” previously said “75SiO_2_-5P_2_O_5_-(15-m)CaO-mCeO_2_”. 

There was an error in the “2.9. In Vivo Wound Healing in a Diabetic Skin Defect Model” section, where the sentence now reading “The skin of wounds were treated with five groups as the gauze (C, control), GelMA (G), 0 Ce-BG/GelMA (0/G), 5 Ce-BG/GelMA (5/G).” previously said “0 Ce-BG/GelMA (0/G), 2 Ce-BG/GelMA (2/G), 5 Ce-BG/GelMA (5/G)”. 

There was an error in the “3.6. The Wound Healing Effects on Diabetic Skin Defect Model In Vivo” section, where the sentence now reading “The wound closure time in groups of 0/G and 5/G were faster than the G and control groups, especially in the 5/G group.” previously said “0/G, 2/G and 5/G”.

The authors apologize for any inconveniences caused and state that the scientific conclusions of the paper are unaffected.

This correction was approved by the Academic Editor. The original publication has also been updated.

## Figures and Tables

**Figure 4:**
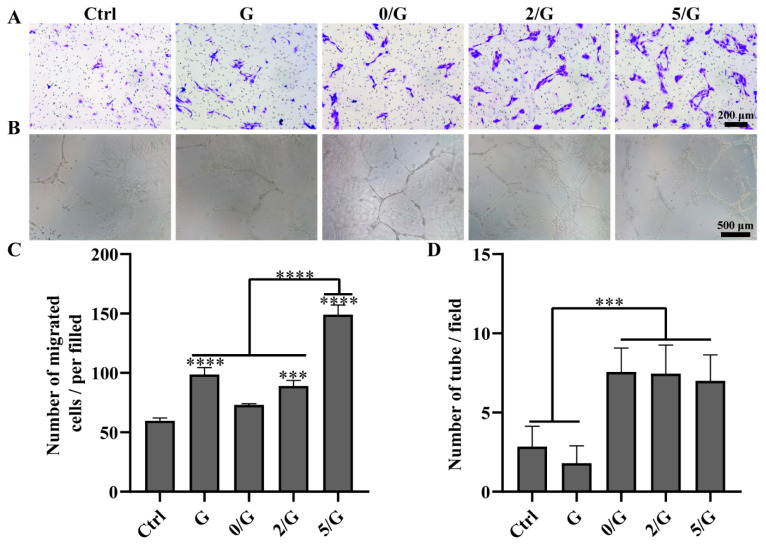
Angiogenic activity evaluation of hydrogels. (**A**) Representative images of HUVECs transwell migration. (**B**) The photographical images of HUVECs tube formation by Matrigel after incubation for 12 h. (**C**) The quantification result of HUVECs migration (*n* = 3). (**D**) The quantification result of HUVECs tube formation (*n* = 3). *** *p* < 0.001, **** *p* < 0.0001.
