# Supplementary material for: Multifunctional Injectable Hydrogel Loaded with Cerium-Containing Bioactive Glass Nanoparticles for Diabetic Wound Healing
Source: Biomolecules. 2021 May 8;11(5):702. doi: 10.3390/biom11050702 (PMC8151889; doi:10.3390/biom11050702)
Supplement: Supplementary file 1 [file biomolecules-11-00702-s001.zip › biomolecules-1205785-supplementary.pdf]

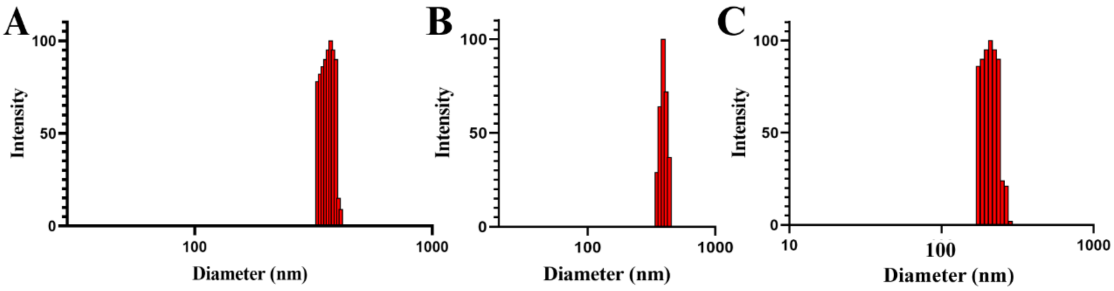

Figure S1. (A–C) Diameter distribution of 0 Ce-BG, 2 Ce-BG and 5 Ce-BG.

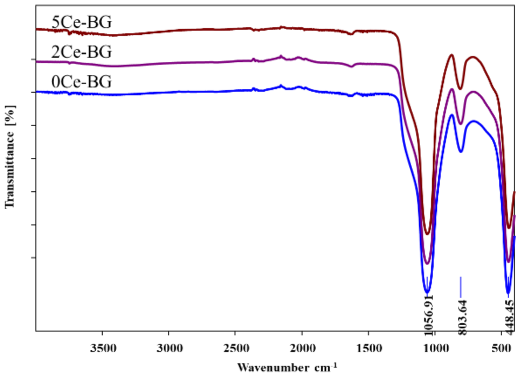

Figure S2. The FTIR spectra of 0 Ce-BG, 2 Ce-BG and 5 Ce-BG.

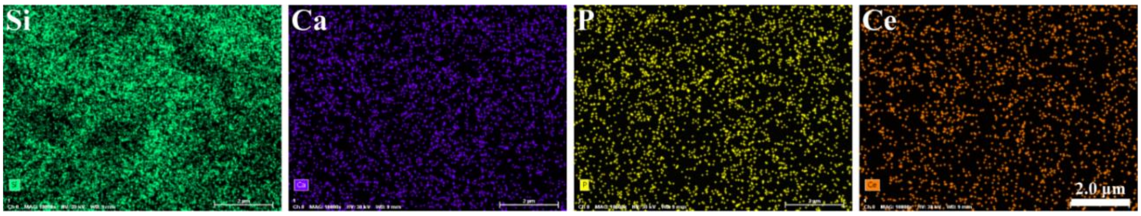

Figure S3. EDX mapping images of 2 Ce-BG.

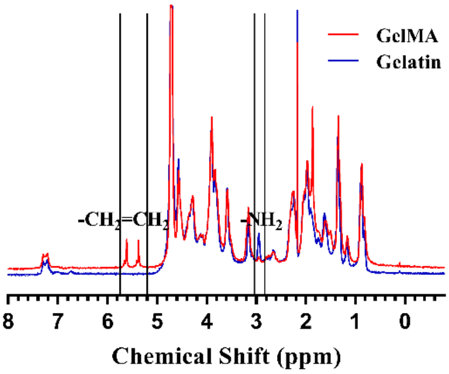

Figure S4. The ^{1}H NMR spectra of GelMA and Gelatin.

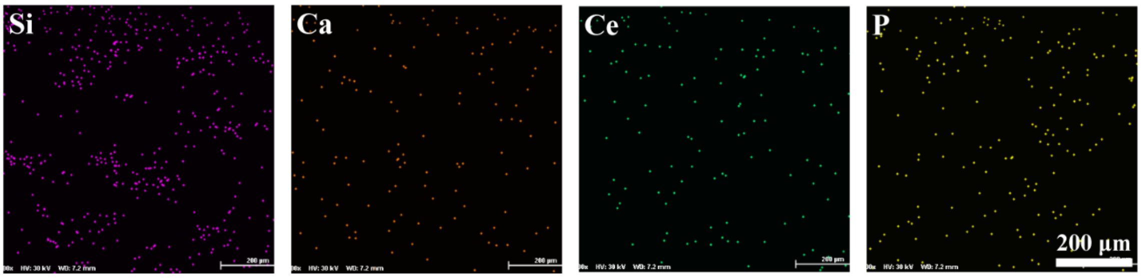

Figure S5. EDX mapping images of 2/G hydrogel.

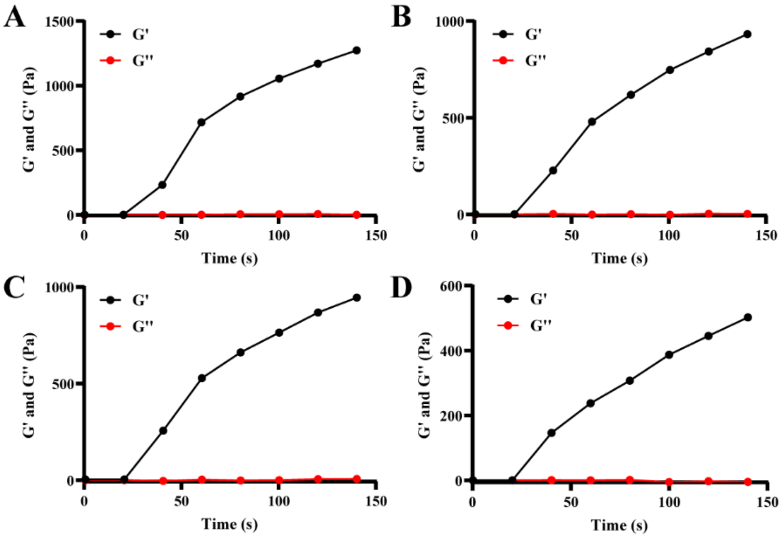

Figure S6. The rheological property of hydrogels, A: G, B: 0/G, C: 2/G, D: 5/G.

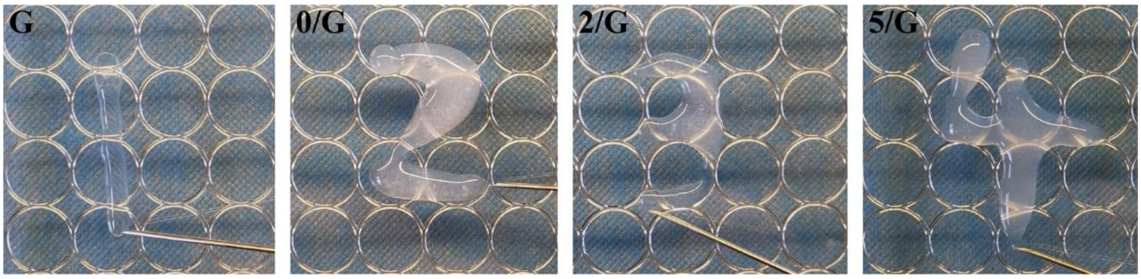

Figure S7. The injectable properties of hydrogel.

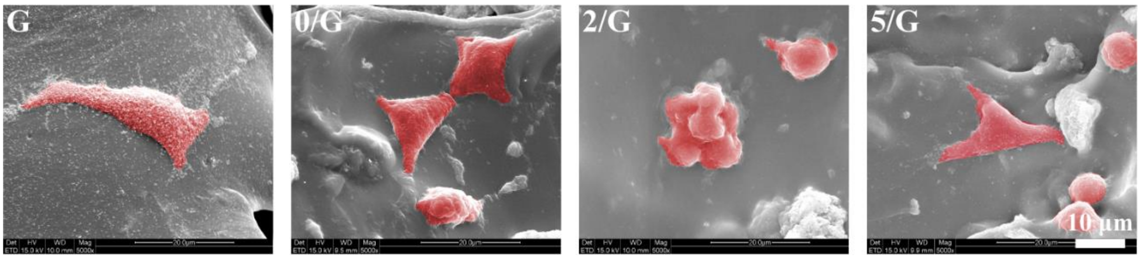

Figure S8. L929 cells adhesion on hydrogels.

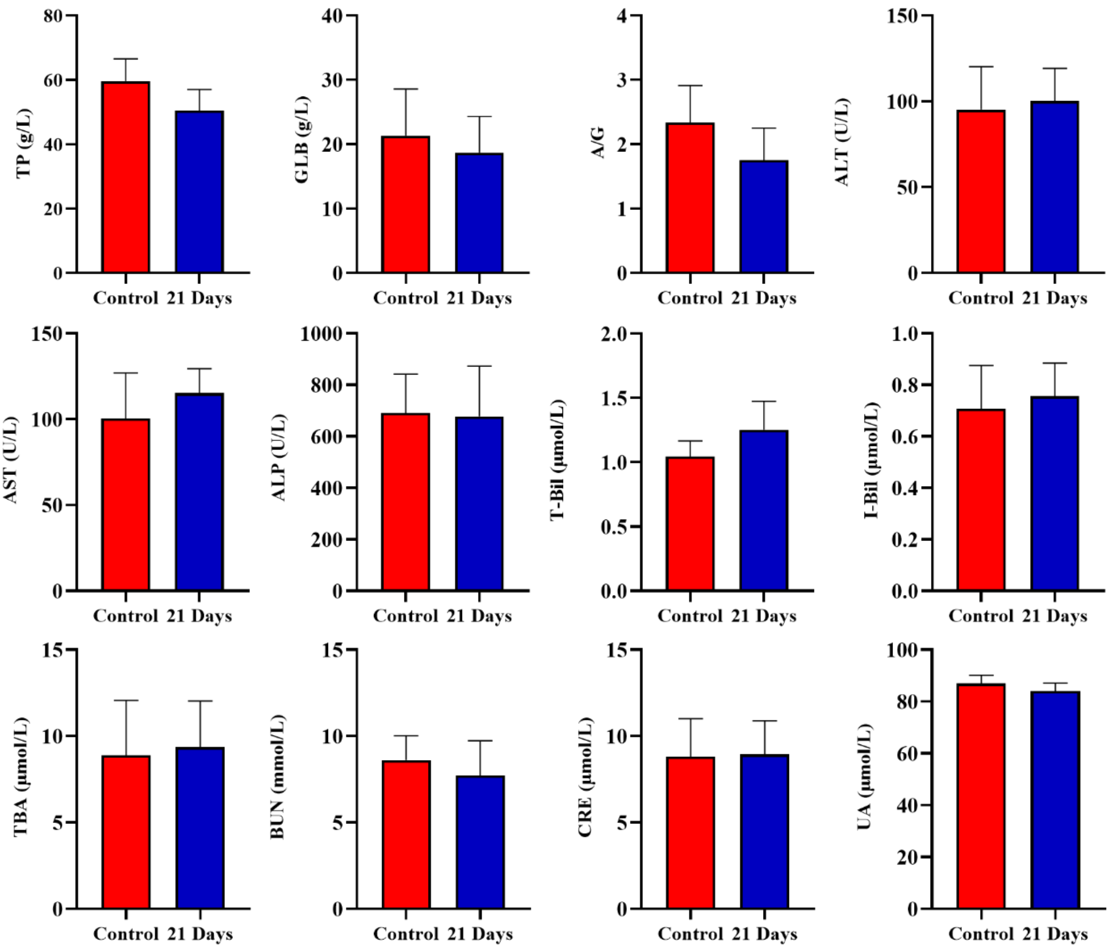

Figure S9. Evaluation of liver and kidney function of rats 21 days after the treatment of the hydrogels **(n = 5)**.
